# Pre-existing morbidity pattern in a Danish testicular cancer cohort: insights beyond the testicular dysgenesis syndrome hypothesis

**DOI:** 10.1093/hropen/hoaf021

**Published:** 2025-04-29

**Authors:** Marie Juul Ornstrup, Agnethe Berglund, Mads Agerbæk, Claus Højbjerg Gravholt

**Affiliations:** Department of Endocrinology and Internal Medicine, Aarhus University Hospital, Aarhus, Denmark; Department of Clinical Medicine, Aarhus University, Aarhus, Denmark; Department of Endocrinology and Internal Medicine, Aarhus University Hospital, Aarhus, Denmark; Department of Clinical Genetics, Aarhus University Hospital, Aarhus, Denmark; Department of Oncology, Aarhus University Hospital, Aarhus, Denmark; Department of Endocrinology and Internal Medicine, Aarhus University Hospital, Aarhus, Denmark; Department of Clinical Medicine, Aarhus University, Aarhus, Denmark; Department of Molecular Medicine, Aarhus University Hospital, Aarhus, Denmark

**Keywords:** testicular cancer, testicular neoplasms, germinoma, gonadal dysgenesis, morbidity, registries, drug prescriptions, cohort study, epidemiology

## Abstract

**STUDY QUESTION:**

Do men diagnosed with testicular cancer (TC) exhibit increased pre-existing morbidity compared to matched controls?

**SUMMARY ANSWER:**

Men with TC had a significantly higher risk of hospital contacts and medicinal use prior to diagnosis compared to controls, reflecting excess morbidity across multiple health domains.

**WHAT IS KNOWN ALREADY:**

The testicular dysgenesis syndrome hypothesis suggests that, e.g. cryptorchidism, poor semen quality, and TC are all symptoms of a fetal gonadal dysgenesis. The association of TC with broader pre-existing morbidity remains unclear.

**STUDY DESIGN, SIZE, DURATION:**

This retrospective, national, registry-based cohort study included 1952 TC patients, identified via the nationwide prospective clinical Danish Testicular Cancer (DATECA) database from 1 January 2013 to 28 February 2019, as well as 19 431 controls.

**PARTICIPANTS/MATERIALS, SETTING, METHODS:**

TC patients were matched with up to 10 randomly selected age-matched males from the background population. None of the controls were at any time registered in the DATECA database or with a TC diagnosis in either The Danish National Patient Register or The National Cancer Register. Hospital contact data and medication prescriptions were evaluated using national registries, categorized by the International Classification of Diseases, 8th edition (ICD-8) prior to 1993 and 10th edition (ICD-10) from 1993 onward, and the Anatomical Therapeutic Chemical (ATC) Classification, using data from birth until TC diagnosis. Negative binomial regression was used to compare ‘Number of hospital contacts’ within each ICD chapter for TC patients versus controls, and stratified Cox regression was used to compare ‘time to first medicinal prescription’ within each ATC-group.

**MAIN RESULTS AND THE ROLE OF CHANCE:**

Prior to the TC diagnosis, the overall risk of hospital contacts was higher among TC patients than controls (incidence rate ratio (IRR)=1.18, CI: 1.13–1.25). IRRs were significantly increased in 11/18 chapters of the ICD-10, including cryptorchism (IRR = 3.24, CI: 2.31–4.52), indeterminate sex (IRR = 13.1, CI: 2.4–70.5), and infertility (IRR = 1.45, CI 1.08–2.01), and there were increased risks of respiratory, digestive, musculoskeletal, and nervous system diseases.

The overall risk of being prescribed any medication was also increased among TC patients before their diagnosis (hazard ratio (HR)=1.28, CI: 1.21–1.34) compared to controls. HRs were significantly increased in 8/14 chapters of the ATC Classification, including the genito-urinary, respiratory, alimentary, musculoskeletal, and nervous system chapters. Risk of androgen prescriptions was not increased, whereas risks of prescription of gonadotropins (HR = 2.90, CI: 1.38–6.08) and medications related to erectile dysfunction (HR = 1.21, CI: 1.00–1.45) were.

**LIMITATIONS, REASONS FOR CAUTION:**

The study is limited by the absence of clinical data, and thus there was no validation of diagnosis codes or prescription indications. However, the Danish national registries are generally recognized as high-quality data sources.

**WIDER IMPLICATIONS OF THE FINDINGS:**

The observed excess morbidity burden challenges the perception that TC males are largely healthy prior to their cancer diagnosis. The morbidity pattern reveals an increased risk of a wide variety of diseases, extending beyond those explained by the TDS hypothesis. These findings underpin that health personnel should be more vigilant regarding the presence of undiagnosed comorbidities and highlight the need for further research into potential shared etiological factors.

**STUDY FUNDING/COMPETING INTEREST(S):**

The study was supported by grants from Aarhus University Hospital Research Fund and Health Research Foundation of Central Denmark Region (Grant number A3488). M.J.O. is an unpaid Medical Specialist Committee member of The Danish Patient Association for Adrenal insufficiency (Addison Foreningen). C.H.G. has received honoraria from Novo Nordisk, Sandoz, and Merck for talks presented on Turner syndrome. The remaining authors have nothing to disclose.

**TRIAL REGISTRATION NUMBER:**

N/A.

WHAT DOES THIS MEAN FOR PATIENTS? This study looked at whether men with testicular cancer had more health problems before their cancer diagnosis compared to men without testicular cancer. Researchers used data from Denmark’s national health records to study men diagnosed with testicular cancer between January 2013 and February 2019. They compared these men to a group of men from the general population who did not have testicular cancer.

The results showed that men with testicular cancer had more health problems across various areas even before their cancer diagnosis, challenging the assumption that these men are generally healthy prior to developing testicular cancer. The results indicate a need for greater awareness of potential undiagnosed health issues in these patients. Gaining a deeper understanding of the burden and types of pre-existing illness in males with testicular cancer may help identify shared risk factors and underlying causes for these comorbidities, potentially leading to opportunities for early detection or preventive interventions.

## Introduction

More than 20 years ago, the ‘Testicular dysgenesis syndrome’ (TDS) was presented as a hypothesis that a cluster of conditions including cryptorchidism, hypospadias, poor semen quality, and testicular cancer (TC), are all symptoms of an underlying fetal gonadal maturation defect (Skakkebaek *et al.*, 2001) and since then, associations between TC and undescended testis (United_Kingdom_Testicular_Cancer_Study_Group, 1994; Cook *et al.*, 2010), hypospadias (Cook *et al.*, 2010; Phillips *et al.*, 2023), and infertility (Møller and Skakkebaek, 1999; Raman *et al.*, 2005) have been well-documented. Childhood inguinal hernias and hydrocele have also been shown to be associated with TC (United_Kingdom_Testicular_Cancer_Study_Group, 1994; Møller and Skakkebaek, 1999) and may, as well, be part of the TDS. Several of the presumed TDS manifestations share common risk factors, for example low birth weight, and they furthermore constitute risk factors for each other (Virtanen *et al.*, 2005). Low birth weight has itself also been suggested as a risk factor of TC (English *et al.*, 2003; Coupland *et al.*, 2004; Stephansson *et al.*, 2011), which could further corroborate the assumption of a common origin due to disruption of gonadal development.

Prenatal exposure to endocrine disruptors, such as pesticides and dermal care products, has been proposed as a likely precipitating cause for development of TDS (Cargnelutti *et al.*, 2021; Faja *et al.*, 2022), but also postnatal exposure to these chemicals, including certain occupational hazards, have in some studies been associated with development of TC (McGlynn and Trabert, 2012; Cargnelutti *et al.*, 2021; Guth *et al.*, 2023). Increased exposure to endocrine disruptors may therefore play a role in the increasing incidence of TC observed over the last four to five decades, particularly in Caucasian populations (Chia *et al.*, 2010; van der Meer *et al.*, 2024).

Originally, it was proposed that the TDS constituted a spectrum, from mild TDS, with primarily impaired spermatogenesis and relatively low risk of TC, through a more severe TDS, with several of the conditions mentioned above, to a very rare severe form of TDS with all features present, including TC and intersex conditions (Skakkebaek *et al.*, 2001). The authors encouraged future epidemiological studies in male reproductive health to do more comprehensive analyses to bring symptoms into a broader context: an approach that could potentially reveal an even greater number of associated diseases. In such terms, a recent Swedish epidemiological study demonstrated an association between neurodevelopmental disorders and testicular seminomas: conditions that are both associated with urogenital problems (Jansson *et al.*, 2023). However, most research of morbidity in males with TC focuses on the period after diagnosis and treatment, rather than pre-existing morbidity.

In this study, we aimed to describe the risk of hospital contacts and medicinal use in a nationwide cohort study of Danish males with TC prior to their TC diagnosis, to increase knowledge of the burden and type of pre-existing morbidity in a broader sense.

## Materials and methods

### Ethical approval

The Danish Data Protection Authority approved the project with the journal number 1-16-02-568-15. Data were accessed via a secure remote access to Statistics Denmark. To avoid any possibility of personal identification of persons, Statistics Denmark prohibits specification of a specific number of cases with a given condition if three or less: thus, it is reported as ≤3.

### Study population and registries

The Danish Civil Registration System was founded in 1968 and since then, every Danish resident has been assigned a unique civil personal 10-digit registration number, allowing accurate data-matching between different registries (Schmidt *et al.*, 2015).

On 1 January 2013, the Danish Multidisciplinary Cancer Group initiated the nationwide prospective clinical Danish Testicular Cancer (DATECA) database in which all Danish males with germ cell cancer, of gonadal as well as extragonadal origin, via the Danish National Patient Registry and/or the Danish Pathology Registry are registered upon diagnosis. The DATECA database includes variables regarding histopathology, cancer stage, prognostic group, and treatment (Daugaard *et al.*, 2016). Throughout this paper, all male germ cell cancers are referred to as TC.

In DATECA, we identified 1952 TC patients from 1 January 2013 to 28 February 2019. Each patient was matched, by Statistics Denmark, the central authority governing the Danish national registries, with up to 10 randomly selected age-matched males from the background population who served as controls (n = 19 480). All controls were alive and living in Denmark at the time of TC diagnosis of their respective case. None of the controls were at any time registered in the DATECA database. However, 49 controls turned out to have been registered with a TC diagnosis in the Danish National Patient Registry and/or The National Cancer Register and were therefore excluded from the study cohort, leaving 19 431 TC-free male controls. Statistics Denmark linked each case and control with data from the following registries.

*The Danish National Patient Registry*: hospital contacts with date and assigned diagnoses were retrieved. From 1977, the registry includes data on all somatic inpatient contacts, and from 1995 all somatic outpatient contacts and psychiatric inpatient and outpatient contacts are also included (Schmidt *et al.*, 2015). Diagnoses are classified according to the International Classification of Diseases, 8th edition (ICD-8) until 1993, and thereafter according to ICD-10 in the registries. For the analyses, we translated ICD-8 diagnoses into ICD-10 diagnoses with inspiration from Pedersen *et al.* (2023).

*The Psychiatric Central Research Register*: data on all psychiatric inpatient contacts in the period from 1970 up until 1995 were retrieved (Mors *et al.*, 2011).

*The Danish National Prescription Registry*: information on all processed medicinal prescriptions since 1995, including medicinal name, date of dispatch, and Anatomical Therapeutic Chemical Classification (ATC) code, were retrieved (Pottegård *et al.*, 2017). This registry includes prescriptions from the primary, as well as the secondary, healthcare system. However, it does not include medicinal use during hospitalization, prescriptions processed by hospital pharmacies, or over-the-counter drugs.

### Statistical analyses

To evaluate overall and cause-specific morbidity identified by hospital contacts, we used complete data on hospital contacts and related diagnosis codes from date of birth or start of registry until TC diagnosis (for controls, the same date as their matched TC patient). To evaluate overall and cause-specific morbidity identified by drug prescriptions, we used complete data on medicinal use from date of birth or start of registry until the TC diagnosis. To minimize the likelihood of including hospital visits and medication usage directly related to an imminent TC diagnosis, all hospital contacts and prescriptions within a 90-day period prior to the TC diagnosis date were excluded. This approach did, however, not include diagnoses related to ‘perinatal disease’ and ‘congenital malformations and genetic disorders’, due to their congenital origin or very early onset. For these diagnoses, time at risk started at birth and ended at death, emigration or the ‘end of study’, whichever came first.

We compared ‘number of hospital contacts’ within each ICD-10 chapter for TC patients versus background population controls, using negative binomial regression, yielding incidence rate ratios (IRR). We compared ‘time to first medicinal prescription’ within each ATC-group using stratified Cox regression, yielding hazard ratios (HRs). CIs were calculated as 95% CIs. Given a significantly increased IRR or HR was observed at the overall level, further analyses were conducted for ICD-10 subchapters and ATC subgroups. ICD-10 chapters R00-R99 and Z00-Z99 were pooled into ‘Other Causes’, and S00 to Y99 were pooled into ‘External cause’.

Since Trisomy 21 is a known risk factor for the development of TC and is associated with significant comorbidity in general, we chose to investigate the impact of this condition on the IRR. After excluding all individuals with Trisomy 21 from the cohort, analyses were repeated for each ICD-10 chapter at the overall level.

To determine whether morbidity differences were mainly influenced by TC cases with congenital malformations and genetic diseases, we assessed the impact of these conditions on IRR. After excluding all individuals with hospital contacts within the ICD-10 chapter ‘Congenital malformations and genetic disorders’ from the cohort, analyses were repeated for each ICD-10 chapter at the overall level.

All analyses were performed using StataCorp. 2023. *Stata Statistical Software: Release 18*. College Station, TX, USA: StataCorp LLC. *P*-values <0.05 were considered statistically significant.

## Results

We included 1952 TC patients and 19 431 age-matched controls. The median age at TC diagnosis was 36.8 (percentile 25&75 (P25–P75): 28.7–46.3) years. Of the TC patients, 59% were diagnosed with seminomas (median age 40.6 years (P25–P75): 33.2–50.1), 37% with non-seminomas (median age 30.0 years (P25–P75): 24.9–38.6), while the subtype was not registered in 4% of cases (median age 37.1 years (P25–P75): 28.8–50.8) (Table 1). According to data in the National Patient Registry, 3 of the 1952 cases were registered with TC prior to their registration in the DATECA database, indicating that they had a relapse or their second TC diagnosis at the time of enrollment in DATECA, thus, their date of TC diagnosis was changed accordingly. There were 404 cases (20.7%) who had disseminated disease at the time of diagnosis, of whom the majority were staged as having ‘good prognosis disease’ prognosis (Table 1).

**Table 1.** Characteristics at time of testicular cancer diagnosis.

	Controls	TC Patients (all)	Seminoma	Non-seminoma	Subtype unknown
**Participants, n (%)**	19 431	1952 (100)	1154 (59.1)	716 (36.7)	82 (4.2)
**Median age at diagnosis, years (P25; P75)**		36.8 (28.7–46.3)	40.6 (33.2–50.1)	30.0 (24.9–38.6)	37.1 (28.8–50.8)
**Localized disease, n (%)**		1548 (79.3)	994 (86.1)	485 (67.7)	69 (84.1)
**Carcinoma *in situ*, n (%)**		58 (3.0)	34 (2.9)	23 (3.2)	1 (1.2)
**Disseminated disease, n (%)**		404 (20.7)	160 (13.9)	231 (32.3)	13 (15.9)
Extragonadal retroperitoneal tumor, n (% of disseminated disease)		36 (8.9)	22 (13.8)	10 (4.3)	4 (30.8)
Extragonadal mediastinal tumor, n (% of disseminated disease)		11 (2.7)	3 (1.9)	7 (3.0)	1 (7.7)
**Prognosis (disseminated disease)**					
Good, n (%)		301 (74.5)	136 (85.0)	157 (68.0)	8 (61.5)
Intermediate, n (%)		60 (14.9)	12 (7.5)	47 (20.3)	1 (7.7)
Poor, n (%)		31 (7.7)	5 (3.1)	23 (10.0)	3 (23.1)
Missing info, n (%)		12 (3.0)	7 (4.4)	4 (1.7)	1 (7.7)

n, number; P25; P75, 25th percentile; 75th percentile; TC, testicular cancer.

### Morbidity identified by hospital contacts

The overall risk of hospital contacts was significantly increased among TC patients compared to controls (IRR = 1.18, CI: 1.13–1.25). Stratified by TC subtype, the overall risk of hospital contacts was significantly higher for patients with seminomas (IRR = 1.24, CI: 1.16–1.32) and for patients with non-seminomas (IRR = 1.13, CI: 1.06–1.22) compared to controls, whereas no difference was observed when comparing TC patients with missing data on subtype with controls (IRR = 0.76, CI: 0.51–1.15).

**Figure 1.**
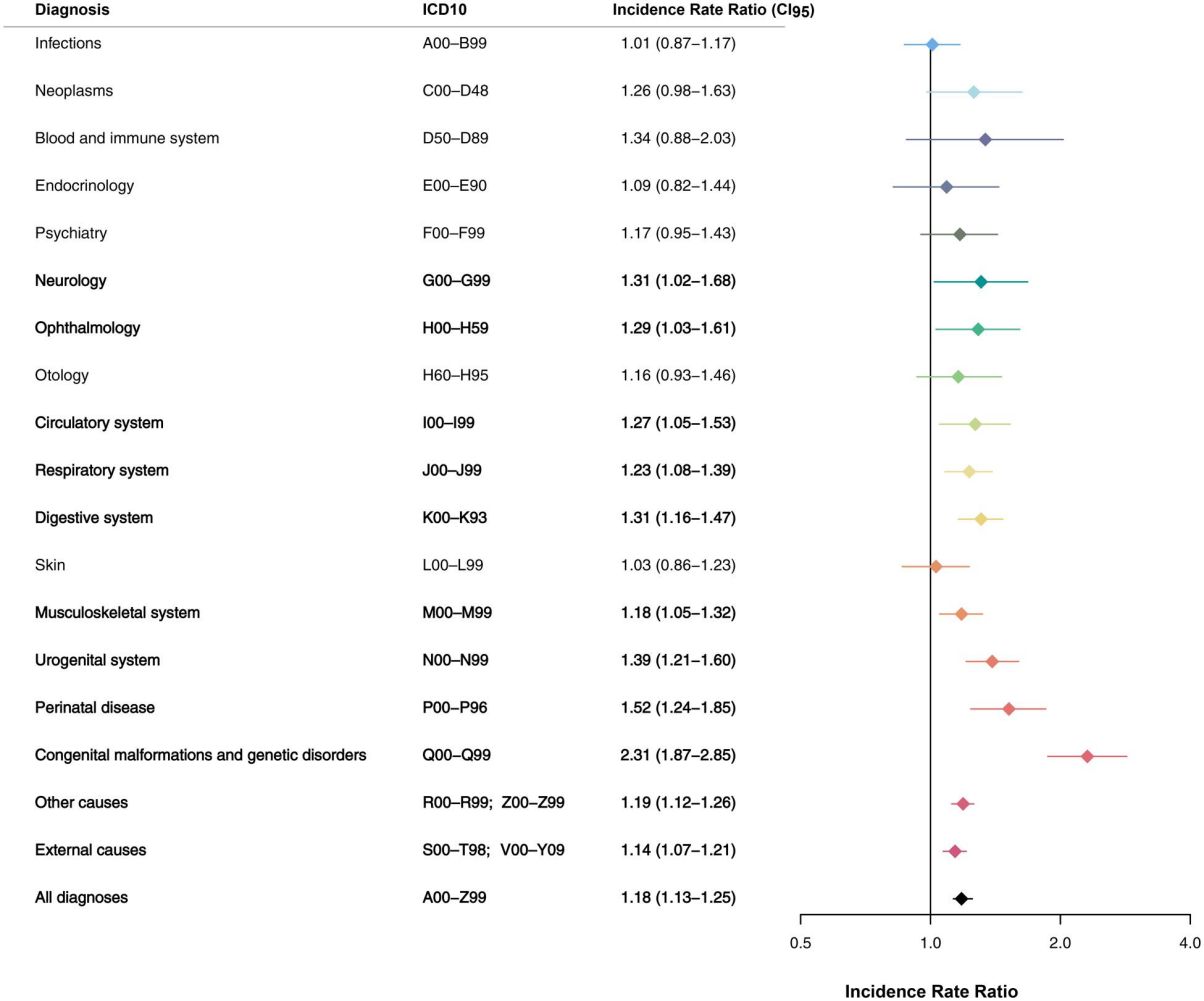
**Morbidity identified by hospital contacts**. Comparison of hospital diagnoses among males with testicular cancer and general population male controls. Diagnoses are divided in diagnostic chapters according to the International Classification of Diseases, 10th edition (ICD10). Negative binomial regression was applied as statistical test to yield incidence rate ratios. Bold text indicates significant results. CI_95_, 95% confidence interval.

When dividing diagnoses into chapters according to ICD-10, TC patients had a significantly increased number of hospital contacts related to diagnoses within the following chapters: neurologic disorders, ophthalmologic disorders, circulatory system disorders, respiratory system disorders, digestive system disorders, musculoskeletal system disorders, urogenital system disorders, perinatal disease, congenital malformations and genetic disorders, ‘other’ diagnoses, and disorders caused by external factors (11 of 18 chapters) (Fig. 1).

**Figure 2.**
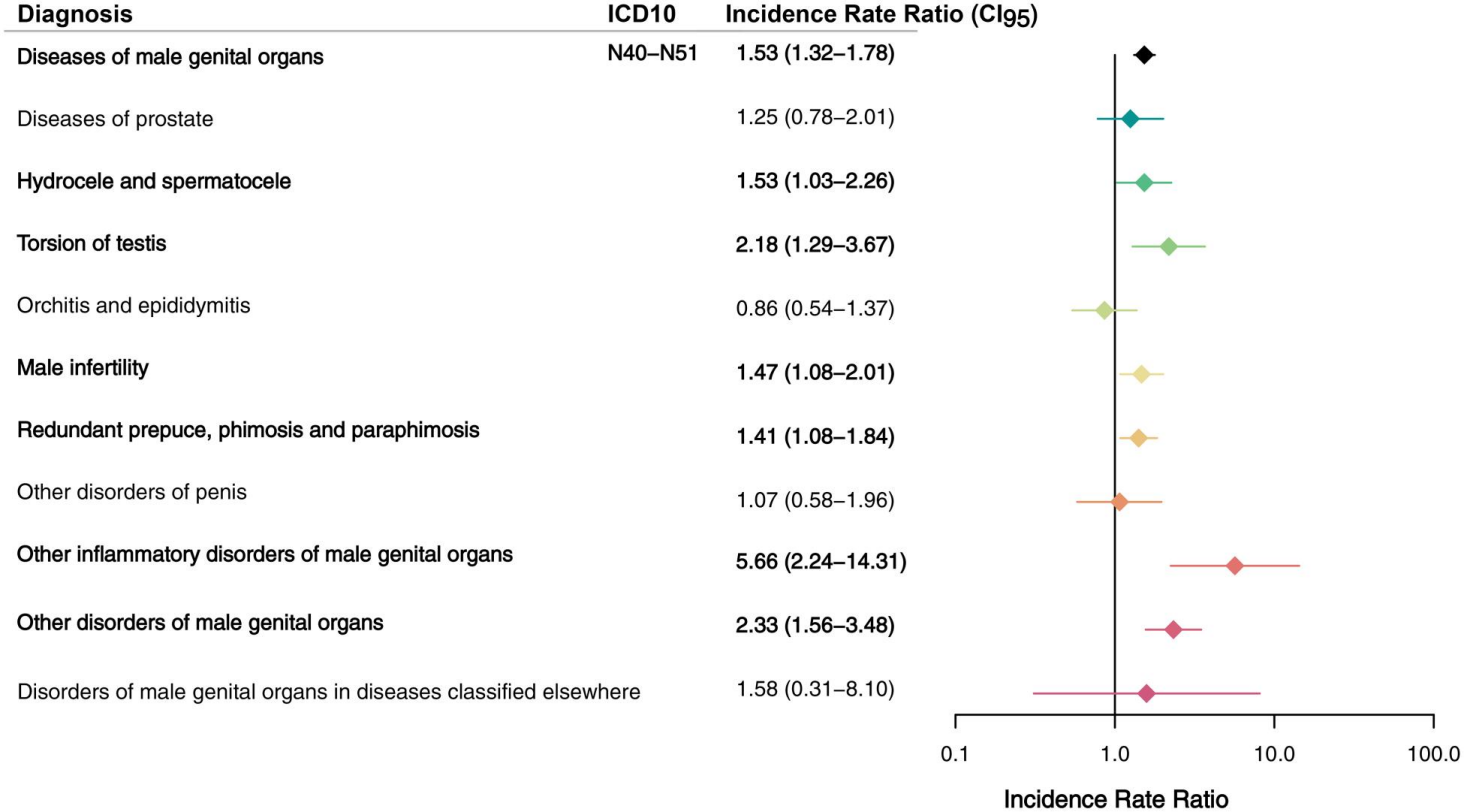
**Morbidity related to diseases of male genital organs**. Comparison of hospital diagnoses among males with testicular cancer and general population male controls within the diagnostic subchapter ‘Diseases of male genital organs’ according to the International Classification of Diseases, 10th edition (ICD10). Negative binomial regression was applied as statistical test to yield incidence rate ratios. Bold text indicates significant results. CI_95_, 95% confidence interval.

**Figure 3.**
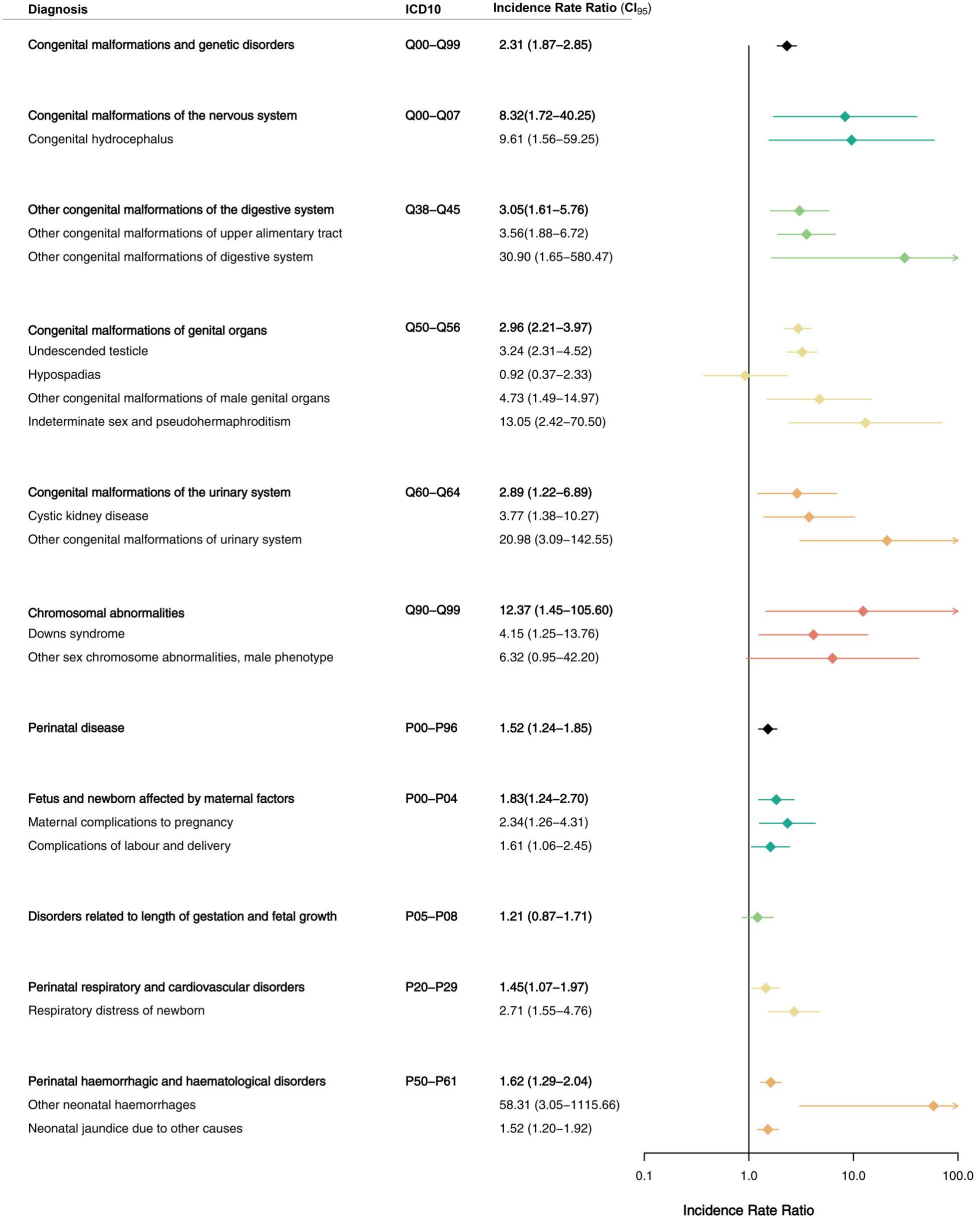
**Morbidity related to congenital malformations, chromosomal abnormalities, and diseases in the perinatal period**. Comparison of hospital diagnoses among males with testicular cancer and general population male controls within the diagnostic chapters ‘Congenital malformations, deformations and chromosomal abnormalities’ and ‘Certain conditions originating in the perinatal period’ according to the International Classification of Diseases, 10th edition (ICD10). Negative binomial regression was applied as statistical test to yield incidence rate ratios. CI_95_, 95% confidence interval.

To provide a more detailed picture of the impact of certain diagnoses on the pre-existing morbidity, each ICD-10 chapter with a significantly increased IRR at the overall level was divided into subgroups. In concordance with the TDS hypothesis, TC patients had a higher risk of hospital contacts with a diagnosis related to male genital organ disease (IRR = 1.53, CI: 1.32–1.78), including an increased risk of male infertility (IRR = 1.47, CI: 1.08–2.01), torsion of testis (IRR = 2.18, CI: 1.29–3.67), hydrocele and spermatocele (IRR = 1.53, CI: 1.03–2.26), redundant prepuce, phimosis and paraphimosis (IRR = 1.41, CI: 1.08–1.84), ‘other inflammatory diseases of male genital organs’ (IRR = 5.66, CI: 2.24–14.31), and ‘other disorders of male genital organs’ (IRR = 2.33, CI: 1.56–3.48) (Fig. 2). Furthermore, TC patients had a higher risk of hernias (IRR = 1.29, CI: 1.06–1.56), congenital malformations of the urinary system (IRR = 2.89, CI: 1.22–6.89), and congenital malformation of the genital organs (IRR = 2.96, CI: 2.21–3.97), herein undescended testis (IRR = 3.24, CI: 2.31–4.52), indeterminate sex (IRR = 13.1, CI: 2.42–70.5), and ‘other congenital malformations of male genital organs’ (IRR = 4.73, CI: 1.49–14.97). The risk of hypospadias was not increased among TC patients (IRR = 0.92, CI: 0.37–2.33) (Fig. 3, Table 2).

**Table 2.** Hospital diagnoses among males with testicular cancer and general population male controls.

Diagnosis	ICD10	IRR (CI_95_)
**Neurology**	G00–G99	**1.31 (1.02–1.68)**
Extrapyramidal and movement disorders	G20–G26	3.51 (1.11–11.10)
Cerebral palsy and other paralytic syndromes	G80–G83	3.84 (1.22–12.10)
**Ophthalmology**	**H00–H59**	**1.29 (1.03–1.61)**
Disorders of optic nerve and visual pathway	H46–H47	3.46 (1.69–7.08)
Disorders of ocular muscles, accommodation, and refraction	H49–H52	1.65 (1.06–2.58)
**Circulatory system**	**I00–I99**	**1.27 (1.05–1.53)**
Pulmonary heart disease and diseases of pulmonary circulation	I26–I28	2.82 (1.11–7.19)
Diseases of veins, lymphatic vessels, and lymph nodes, not elsewhere classified	I80–I89	1.43 (1.09–1.88)
**Respiratory system**	**J00–J99**	**1.23 (1.08–1.39)**
Influenza and pneumonia	J09–J18	1.35 (1.06–1.70)
Chronic lower respiratory diseases	J40–J49	1.48 (1.10–1.99)
Lung disease due to external agents	J60–J79	3.53 (1.05–11.89)
Suppurative and necrotic conditions of the lower respiratory tract	J85–J86	6.35 (2.65–15.22)
**Digestive system**	**K00–K93**	**1.31 (1.16–1.47)**
Hernia	K40–K46	1.29 (1.06–1.56)
**Musculoskeletal system**	**M00–M99**	**1.18 (1.05–1.32)**
**Other causes**	**R00–R99; Z00–Z99**	**1.19 (1.12–1.26)**
**External causes**	**S00–T98; V00–Y09**	**1.14 (1.07–1.21)**

Incidence rate ratios (IRRs) for chapters with significant findings, beyond those shown in Fig. 2 or Fig. 3, according to the 10th edition of the International Classification of Diseases (ICD10). Negative binomial regression was applied as a statistical test to yield IRRs. Bold text indicates overall ICD10 chapter. CI_95_, 95% confidence interval.

In terms of perinatal diseases, TC patients had an increased risk of perinatal respiratory and cardiovascular disorders (IRR = 1.45, CI : 1.07–1.97), perinatal hemorrhagic and hematologic disorders (IRR = 1.62, CI: 1.29–2.04), and diagnoses encompassed by the subchapter ‘Fetus and newborn affected by maternal factors’ (IRR = 1.83, CI : 1.24–2.70). TC patients did not have an increased risk of disorders related to length of gestation and fetal growth (IRR = 1.21, CI: 0.87–1.71) (Fig. 3).

The risk of several circulatory and respiratory conditions was increased among TC patients, as were diagnoses related to disorders affecting the optic nerve and ocular muscles, accommodation, and refraction (Table 2).

Concerning congenital malformations, TC patients had an increased risk of malformations not previously related to TDS, including the nervous system (IRR = 8.32, CI: 1.72–40.25) and digestive system (IRR = 3.05, CI: 1.61–5.76). Also increased was the risk of chromosomal abnormalities (IRR = 12.37, CI: 1.45–105.6), of which Trisomy 21 was dominant (IRR = 4.15, CI: 1.25–13.76), while ‘other sex chromosome abnormalities, male phenotype’, including Klinefelter Syndrome, did not reach statistical significance (IRR = 6.32, CI: 0.95–42.20) (Fig. 3).

A total of 17 individuals in this entire cohort had a diagnosis of Trisomy 21 (6 cases and 11 controls). After excluding all individuals with Trisomy 21 from the cohort, analyses were repeated for each chapter at the overall level. This did not result in any significant change of the IRR in any of the chapters (data not shown).

To determine whether morbidity differences were driven mainly by the TC cases with congenital malformations and genetic diseases, we excluded all individuals with hospital contacts within the ICD-10 chapter ‘Congenital malformations and genetic disorders’ and repeated analyses (n = 1706 cases and 17 930 controls). This adjustment rendered the IRR non-significant for the chapters ‘Neurology’ (IRR = 1.10, CI: 0.85–1.43) and ‘Opthalmology’ (IRR = 1.14, CI: 0.91–1.43), while leaving all other ICD-10 chapters unaffected (data not shown). A similar pattern was observed when we additionally excluded individuals with hospital contacts within the ICD-10 chapter ‘Perinatal disease’ (data not shown).

### Morbidity identified by drug prescriptions

The overall risk of being prescribed any medication was significantly increased among TC patients compared to controls (HR = 1.28, CI: 1.21–1.34). Stratified by TC subtype, patients with seminomas had a 30% higher risk than controls (HR = 1.30, CI: 1.22–1.39), and patients with non-seminomas had a 38% higher risk (HR = 1.38, CI: 1.27–1.51). The opposite trend was evident for TC patients with missing data on subtype as they had a significantly lower risk of being prescribed any medication than their controls (HR = 0.50, CI: 0.37–0.67).

**Figure 4.**
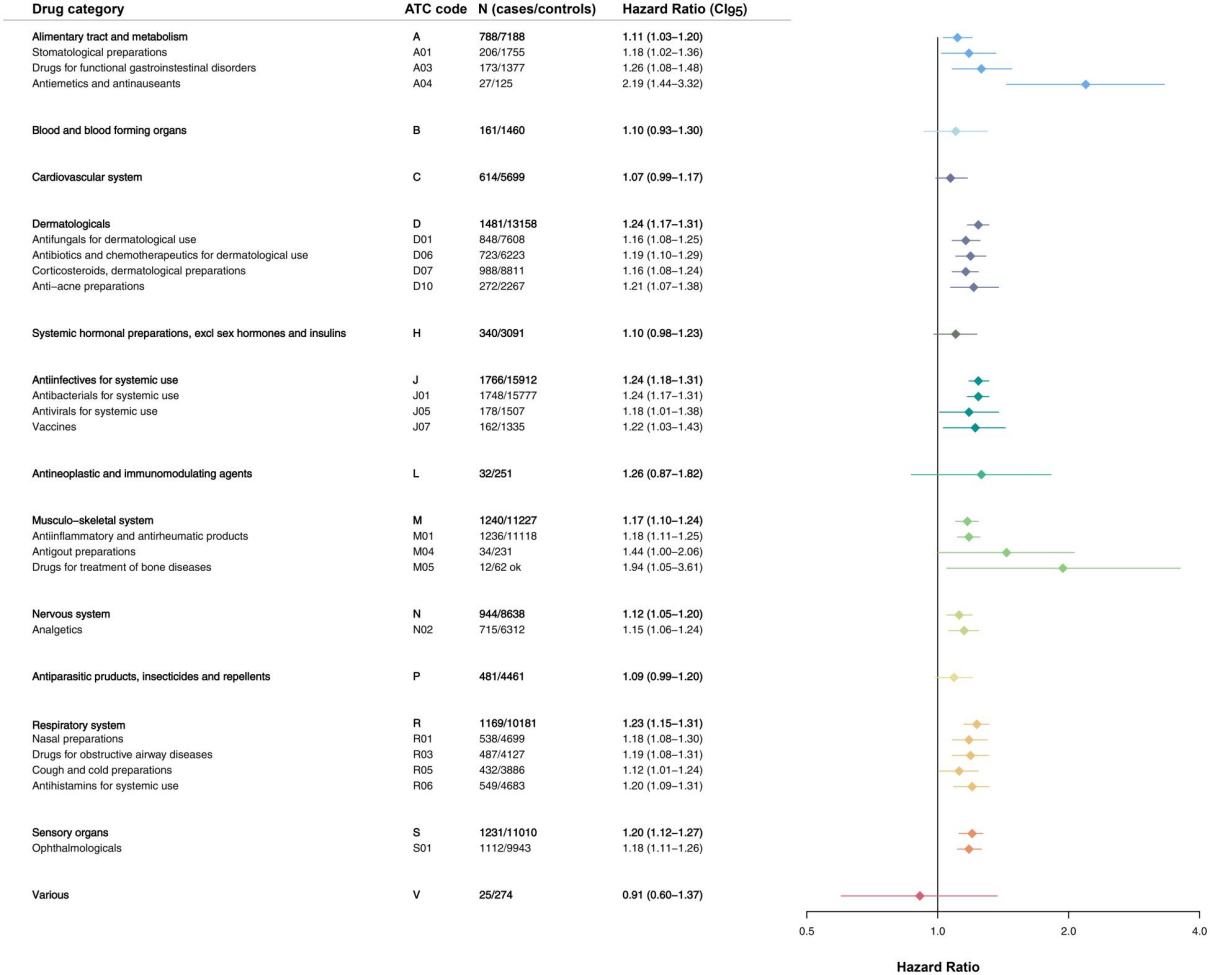
**Morbidity identified by drug prescriptions**. Comparison of prescribed medication among males with testicular cancer and general population male controls. Prescribed medication is divided in chapters according to the Anatomic Therapeutic Classification system (ATC). Stratified Cox regression was applied as statistical test to yield hazard ratios. N, number; CI_95_, 95% confidence interval.

**Figure 5.**
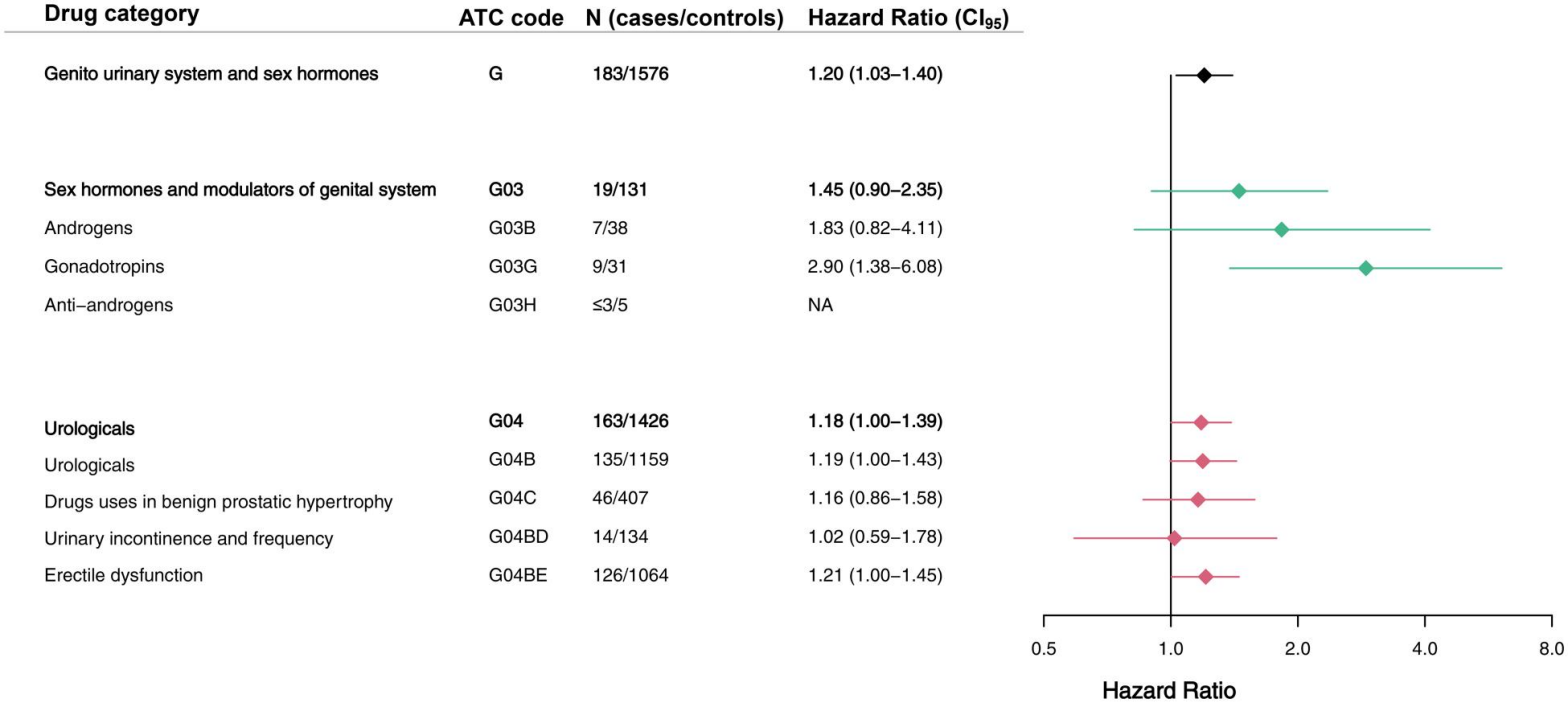
**Drug prescriptions related to the genito-urinary system and sex hormones**. Comparison of prescribed medication among males with testicular cancer and general population male controls within the drug category ‘Genito-urinary system and sex hormones’, according to the Anatomic Therapeutic Classification system (ATC). Stratified Cox regression was applied as statistical test to yield hazard ratios. N, number; CI_95_, 95% confidence interval.

Dividing prescriptions in chapters according to the ATC classification, a significantly increased risk was observed for TC patients within the following 8 of 14 chapters: alimentary tract and metabolism, dermatologicals, genito-urinary system and sex hormones, anti-infectives for systemic use, musculoskeletal system, nervous system, respiratory system, and sensory organs (Figs 4 and 5).

To provide a more detailed picture of pre-existing co-morbidity requiring medicinal use, each ATC chapter with a significantly increased HR at the overall level was divided into subgroups.

The risk of prescriptions for functional gastrointestinal disorders (HR = 1.26, CI: 1.08–1.48), antiemetics (HR = 2.19, CI: 1.44–3.32), and stomatological preparations (HR = 1.18, CI: 1.02–1.36) was significantly increased for TC patients compared to controls. Likewise, was the risk of prescription of systemic antibiotics (HR = 1.24, CI: 1.17–1.31), systemic antivirals (HR = 1.18, CI: 1.01–1.38) and vaccines (HR = 1.22, CI: 1.03–1.43). TC patients also had an increased risk of prescription for obstructive airway diseases (HR = 1.19, CI: 1.08–1.31), cough and cold preparations (HR = 1.12, CI: 1.01–1.24), and systemic anti-histamines (HR = 1.20, CI: 1.09–1.31) in addition to an increased risk of a variety of dermatological preparations (antifungals, antibiotics and chemotherapeutics, corticosteroids, and anti-acne preparations), medications for the musculoskeletal system (anti-inflammatory and anti-rheumatic products, anti-gout preparations, and medications for treatment of bone diseases), medications for the eyes (HR = 1.18, CI: 1.11–1.26), and analgetics (HR = 1.15, CI: 1.06–1.24) (Fig. 4).

TC patients did not have a higher risk of prescription of androgens prior to the TC diagnosis (HR = 1.83, CI: 0.82–4.11), however, they did have a higher risk of being prescribed gonadotropins (HR = 2.90, CI: 1.38–6.08) and medications related to erectile dysfunction (HR = 1.21, CI: 1.00–1.45) (Fig. 5).

### Temporal changes in morbidity pattern

The risk of hospital contacts and medication prescriptions 5 and 10 years before the TC diagnosis largely mirror the patterns observed 90 days before diagnosis (Table 3). This suggests that disease occurrence and distribution remained relatively stable over time, with an early onset.

**Table 3.** Temporal changes in morbidity.

	Incidence rate ratio (CI_95_)		
Number of hospital contacts	Ten years before TC diagnosis	Five years before TC diagnosis	Ninety days before TC diagnosis
All diagnoses (ICD10)	1.20 (1.14–1.27)	1.19 (1.13–1.25)	1.18 (1.13–1.25)
Neurology (G00–G99)	1.26 (0.88–1.80)	1.22 (0.90–1.64)	1.31 (1.02–1.68)
Opthalmology (H00–H59)	1.48 (1.13–1.92)	1.38 (1.09–1.73)	1.29 (1.03–1.61)
Circulatory system (I00–I99)	1.36 (1.04–1.79)	1.34 (1.07–1.68)	1.27 (1.05–1.53)
Respiratory system (J00–J99)	1.20 (1.04–1.39)	1.21 (1.06–1.39)	1.23 (1.08–1.39)
Digestive system (K00–K93)	1.32 (1.15–1.52)	1.35 (1.18–1.53)	1.31 (1.16–1.47)
Musculoskeletal system (M00–M99)	1.16 (1.00–1.34)	1.19 (1.05–1.35)	1.18 (1.05–1.32)
Urogenital system (N00–N99)	1.25 (1.03–1.52)	1.30 (1.10–1.52)	1.39 (1.21–1.60)
Perinatal disease (P00–P96)	1.54 (1.25–1.89)	1.53 (1.25–1.87)	1.52 (1.24–1.85)
Congenital malformations and genetic disorders (Q00–Q99)	2.28 (1.81–2.89)	2.24 (1.79–2.80)	2.31 (1.87–2.85)

	Hazard ratio (CI_95_)		
Time to first drug prescription	Ten years before TC diagnosis	Five years before TC diagnosis	Ninety days before TC diagnosis
All drug prescriptions (ATC)	1.26 (1.19–1.32)	1.27 (1.20–1.34)	1.28 (1.21–1.34)
Alimentary tract & metabolism (A)	1.06 (0.96–1.17)	1.08 (0.99–1.17)	1.11 (1.03–1.20)
Dermatologicals (D)	1.23 (1.15–1.31)	1.24 (1.17–1.31)	1.24 (1.17–1.31)
Genitourinary system & sex hormones (G)	1.40 (1.09–1.80)	1.27 (1.05–1.54)	1.20 (1.03–1.40)
Antiinfectives for systemic use (J)	1.21 (1.14–1.28)	1.23 (1.17–1.30)	1.24 (1.18–1.31)
Musculoskeletal system (M)	1.10 (1.02–1.19)	1.16 (1.08–1.23)	1.17 (1.10–1.24)
Nervous system (N)	1.10 (0.99–1.21)	1.13 (1.05–1.23)	1.12 (1.05–1.20)
Respiratory system (R)	1.22 (1.13–1.31)	1.24 (1.16–1.33)	1.23 (1.15–1.31)
Sensory organs (S)	1.18 (1.10-1.27)	1.19 (1.12–1.27)	1.20 (1.12–1.27)

Morbidity, identified by number of hospital contacts, presented as incidence rate ratio (IRR) 10 years, 5 years, and 90 days before the testicular cancer diagnosis. Morbidity, identified by time to first drug prescription, presented as hazard ratio (HR) 10 years, 5 years, and 90 days before the TC diagnosis. Negative binomial regression was applied as statistical test to yield IRRs, and stratified Cox regression to yield HRs. ATC, Anatomical Therapeutic Chemical Classification; CI_95_, 95% confidence interval; ICD10, International Classification of Diseases, 10th edition; TC, testicular cancer.

## Discussion

### Excess pre-existing morbidity across multiple health domains

This population-based study, which encompasses an entire national cohort of TC patients, shows that TC patients exhibit an increased and very diverse comorbidity pattern already before receiving their TC diagnosis, when compared to age-matched background population male controls. The pre-existing comorbidity included anticipated TDS-related diseases, but we also uncovered novel and hitherto unknown disease associations likely underscoring the importance and broader implications of pre-existing morbidity in TC patients.

We found that TC patients prior to their TC diagnosis both had a significantly increased overall hospital-related morbidity, and a significantly increased overall drug-related morbidity compared to controls. While many studies have reported increased morbidity after TC treatment, either as a direct consequence of surgery, chemotherapy, or radiation, or due to potential psychological issues, the literature provides only little data on pre-existing morbidity in a broad sense (Fung *et al.*, 2015; Kerns *et al.*, 2018; Schepisi *et al.*, 2019).

### TDS-related pre-existing morbidity

Our results partly support the TDS hypothesis by showing an increased risk of hospital contacts with diagnoses related to congenital malformations of genital organs, including undescended testis and indeterminate sex. Interestingly, however, the risk of hypospadias was not increased. In a Swedish population-based cohort study, Phillips *et al.* (2023) showed an increased risk of seminomas in males born with hypospadias, while an earlier meta-analysis of perinatal variables in relation to TC concluded that hypospadias could not be analyzed due to the few patients exposed (Cook *et al.*, 2010). The accuracy of the registration of hypospadias in the Danish National Patient Registry has previously been validated, and found to be of overall very high validity (Arendt *et al.*, 2017), Thus, our results challenge the perception of an association between hypospadias and TC, however, further studies are warranted.

Our data showed an increased risk of infertility, torsion of the testis, hydrocele and spermatocele, redundant prepuce, phimosis, and paraphimosis, among other disorders of male genital organs. Fertility issues prior to a TC diagnosis are generally acknowledged, and pre-existing low semen quality, lower number of children than expected, and increased risk of having an infertility diagnosis have been reported in males whom are later diagnosed with TC (Møller and Skakkebaek, 1999; Jacobsen *et al.*, 2000; Baker *et al.*, 2005). However, torsion of the testis and hydrocele have not. The validity of these diagnoses in the National Patient Registry is uncertain (Schmidt *et al.*, 2015), and we therefore cannot rule out that these findings stem from initial misdiagnosis of a TC. However, as we discarded all diagnoses 90 days prior to the TC diagnosis, we believe to have reduced this risk substantially. On the other hand, Kafka *et al.* (2020) found no incidental TCs in pre-surgical ultrasound in 157 males with hydrocele (aged 20–60 years old), arguing against such an association, although this study was not large.

In contrast to what the TDS hypothesis suggests, Danish TC patients did not have a higher risk of testicular hypogonadism, nor prescription of androgens before the TC diagnosis and treatment. They did, however, exhibit a higher risk of being prescribed gonadotropins (HR = 2.90, CI: 1.38–6.08) and medications related to erectile dysfunction. Age at first prescription of gonadotropin, predominantly hCG, ranged from the age of 3 years old to mid-30s (data not shown), which likely implies anything from attempts to descend testicles, tests for hypogonadism, need for puberty induction, and infertility treatment. Based on the present registry data we can, however, only speculate.

Some studies suggest a link between TDS features, including TC, and adverse birth outcomes. A Danish study found an increased risk of cryptorchidism and hypospadias with lower birth weight, independent of gestational age (Weidner *et al.*, 1999), and a US study reported a higher risk of seminomas in term infants with low birth weight (<2500 g), though without a gestational age association (English *et al.*, 2003). Other studies claiming supporting evidence of an association between TC and low birth weight and preterm birth, however, failed to reach statistical significance (Coupland *et al.*, 2004; Stephansson *et al.*, 2011). A meta-analysis of perinatal variables in relation to TC found evidence that cryptorchidism and inguinal hernia are associated with increased risk of TC, and tentative evidence that low birth weight and preterm birth are associated with TC (Cook *et al.*, 2010). Here we cannot confirm any association between TC and ‘disorders of newborn related to length of gestation and fetal growth’. However, several perinatal diagnoses were overrepresented among TC patients compared to controls, herein perinatal respiratory and cardiovascular disorders, perinatal hemorrhagic and hematologic disorders (including jaundice), and perinatal diagnoses within the subchapter ‘Fetus and newborn affected by maternal factors’, with possible relation to intrauterine growth retardation and/or preterm birth. From The Danish Medical Birth Register, we were able to retrieve data for a subgroup of our cohort on birth weight (n = 10 770) and gestational age (n = 7771), in which we found no differences between TC patients and controls in either birth weight or gestational age, regardless of TC subtype (data not shown).

### Novel pre-existing disease associations extending beyond TDS

Besides findings related to the TDS hypothesis, this study also provides novel findings of hitherto unknown disease associations and a surprisingly substantial burden of pre-existing comorbidity. We found a greater risk of hospital contacts within 11 of 18 ICD-10 chapters, and a greater risk of drug prescriptions within 8 of 14 ATC groups, among cases prior to their TC diagnosis. For example, increased risk of congenital malformations of the nervous system and digestive system, non-congenital diseases of the nervous system and the eye, as well as prescriptions related to the alimentary tract system and ophthalmologicals. The same applies to diseases within the respiratory and circulatory system, and use of anti-infective drugs, respiratory system drugs, in addition to several dermatological preparations. Very few studies have focused on non-TDS-related pre-existing morbidity, providing limited opportunity to discuss these novel findings. Jansson *et al.* (2023) proposed an association between neurodevelopmental disorders and testicular seminoma, with a small, but significantly, increased risk of seminoma among males with a history of a neurodevelopmental disorder. We could not confirm this increased risk from either hospital contacts or medicinal use data. Al-Jebari *et al.* (2018, 2019) described a higher risk of congenital malformations overall among children of fathers with a later TC diagnosis, compared with offspring fathered by males without TC, suggesting shared pathogenetic factors. Our study results suggest TC as a biomarker of general male health. The consistently increased risk of hospital contacts and medication prescriptions both a decade before and immediately prior to the TC diagnosis suggests that disease occurrence and distribution remain stable over time, with an early onset. We anticipated early diagnoses of TDS-related conditions, whereas we expected non-TDS-related findings to increase closer to the TC diagnosis. Given this, we speculated about whether the presumably most severe TDS cases were the main drivers of our findings. However, even after excluding individuals with congenital malformations and genetic diseases, morbidity across all ICD-10 chapters, except for ‘Neurology’ and ‘Ophthalmology’, remained unchanged. Thus, the remaining TC cohort still exhibited higher pre-existing morbidity compared to controls, also in most non-TDS-related conditions.

Another possible manifestation of TDS, namely poor semen quality, has also been proposed as a biological marker of long-term morbidity and mortality (Jensen *et al.*, 2009; Latif *et al.*, 2017). Latif *et al.* (2017) described a clear association between low sperm count and all-cause hospitalizations, as well as cardiovascular disease, comparable to our findings regarding TC and associated morbidity patterns.

### Pre-existing morbidity according to TC subtype

Overall hospital-related morbidity and medicinal use were to some extent dependent on the subtype of TC. The subgroup with seminomas was older and a lower proportion had disseminated disease at the time of diagnosis compared to the subgroup with non-seminomas. Despite more localized disease, the pre-existing hospital-related morbidity burden seemed slightly higher compared to non-seminomas, but medicinal use was lower. The results from the small group of patients with unknown TC subtype deviated from the other TC subtypes. They did not have an increased risk of hospital contacts compared to controls, and they had a decreased risk of medicinal use. These results surprised us. We speculate that the ‘unknown subtype’ group may have been less inclined to seek healthcare, though their proportion of disseminated disease at diagnosis did not suggest a diagnostic delay. However, more had extragonadal disease and were staged with poor prognosis. Some with unknown TC subtype could represent patients with a large tumor burden, diagnosed clinically and through serology, not undergoing initial orchiectomy or biopsy, leading to the lack of a histopathological diagnosis. The database does not provide information on why the subtype was undetermined, leaving our speculations unsupported.

### Strengths and limitations

A major strength of this study is the nationwide approach, based on the DATECA database containing complete data on all males diagnosed with TC in Denmark in the study period, minimizing selection bias substantially. The generally high validity of the DATECA database, due to its administrative and prospective collection of data, is another major strength of the study (Daugaard *et al.*, 2016; Clinical Quality registries, Copenhagen, Denmark, 2022). Moreover, by including data on prescribed medications, we were able to detect diseases even if they were managed by a primary healthcare provider and did not lead to a hospital encounter (e.g. chronic respiratory disease or mild infectious disease). Additionally, the close matching with controls enabled a strict comparison with the general population. A limitation of the present study is the lack of clinical data, giving us no opportunity to validate the diagnosis codes or indication for prescriptions. Under-reporting of certain disorders cannot be ruled out either. However, in general, the Danish national registries are considered high-quality databases (Thygesen and Ersbøll, 2014; Schmidt *et al.*, 2015; Schmidt *et al.*, 2019), partly due to the Danish universal tax-funded healthcare system with residency-based entitlement to healthcare, government-maintained nationwide registries with longitudinal and lifelong collected data, and the unique civil personal registration number allowing accurate data-matching between registries.

### Perspectives

Gaining a deeper understanding of the burden and types of pre-existing morbidities in males with TC may help identify shared risk factors and underlying causes for these comorbidities, potentially leading to opportunities for early detection or preventive interventions. The data presented clearly indicate that TC patients are more fragile throughout life than previously thought, and the pre-existing excess morbidity was related to multiple conditions, not all directly explainable from the TDS hypothesis. Whether this could be a common feature in other cancer cohorts remains unknown and calls for similar nationwide register-based cohort studies in other cancer types.

## Conclusion

We report a systematic investigation of pre-existing morbidity in an unselected nationwide cohort of TC males using complete data on hospital diagnoses and medicinal use. The study highlights a substantial excess morbidity burden before the TC diagnosis, challenging the perception that TC males are largely healthy prior to their cancer diagnosis. The morbidity pattern reveals an increased risk of a wide variety of diseases, not all directly explainable from the TDS hypothesis. Based on the present data, we cannot draw firm conclusions on the underlying cause of this excess morbidity, however, our findings underpin that health personnel should be more vigilant regarding the presence of undiagnosed comorbidities than previously presumed when clinically ascertaining males with TC.

## Data Availability

Due to strict protection of privacy under Danish law, individual datasets are not publicly available.
